# Traumatic Anterior Tibial Artery Pseudoaneurysm: A Case Report

**DOI:** 10.5811/cpcem.1458

**Published:** 2023-11-08

**Authors:** Aaron Thomas, Ga-ram Han, Ina Soh, James Komara

**Affiliations:** * Mayo Clinic, Department of Emergency Department, Phoenix, Arizona; † Mayo Clinic, Department of Vascular Surgery, Phoenix, Arizona

**Keywords:** pseudoaneurysm, ultrasound, compartment syndrome, case report

## Abstract

**Introduction:**

Traumatic pseudoaneurysms of the limbs are rare, with few cases described in vascular literature. Treatment is variable and dependent upon presentation and impact on local anatomy affected. Rapid assessment can be performed with ultrasound and assist in treatment decisions. We describe a case of traumatic anterior tibial artery pseudoaneurysm, which was rapidly identified with point-of-care ultrasound leading to definitive surgical management.

**Case Report:**

A 37-year-old female presented to the emergency department for evaluation of right lower extremity pain and swelling following an exercise session with weighted squats and thigh abductor machines. She was found to have an anterior tibial artery pseudoaneurysm on point-of-care ultrasound, later confirmed with formal ultrasound as well as angiography, and was admitted for surgical management.

**Conclusion:**

Traumatic pseudoaneurysms can rapidly be differentiated from other mass lesions and contributors to compartment syndrome using point-of-care ultrasound.

CPC-EM Capsule Summary What do we already know about this clinical entity?

*Arterial pseudaneurysms of the limbs are rare and are usually the result of trauma. They are potentially limb-threatening.*

What makes this presentation of disease reportable?

*Point-of-care ultrasound rapidly identified pulsatile pseudoaneurysm, avoiding bedside compartment pressure measurement that could have caused hemorrhage.*

What is the major learning point?

*Point-of-care ultrasound (POCUS) can rapidly characterize vascular sources of limb swelling, including pseudaneurysm.*

How might this improve emergency medicine practice?

*Continued POCUS practice for emergency clinicians will enable them to feel comfortable making limb- saving decisions.*

## INTRODUCTION

Arterial pseudoaneurysm is defined as a vascular wall abnormality resulting in blood collection in adjacent extraluminal space.^1^ Traumatic pseudoaneurysms of the limbs are quite rare, and there is a paucity of literature to standardize work-up in the emergency department (ED) setting. There are few case reports on tibial artery pseudoaneurysms with the majority related to prior trauma or infectious etiologies. Spontaneous occurrences are essentially unreported. Multiple studies in the surgical literature have demonstrated various repair techniques. However, there is a paucity of data in the emergency medicine literature regarding identification on acute presentation, potential emergent complications, and options for management.^2^ ^,^ ^3^ Lower extremity pseudoaneurysms are often found in the femoral vasculature with trials of compression therapy favored before surgical intervention based on the patient’s clinical presentation.^4^ ^,^ ^5^

## CASE REPORT

A 37-year-old female presented to the ED for evaluation of right lower extremity pain and swelling immediately following an exercise session about 24 hours earlier that involved squat motion and hip abduction with maximum load of 40 pounds. She additionally complained of decreased sensation to the dorsum of her right foot. These symptoms progressed through the next 24 hours, and the patient noticed a worsening expanding mass to the lateral aspect of her proximal right lower leg. Eight months prior to presentation, the patient had been in a motor vehicle collision that resulted in an open right tibial shaft fracture and fibular fracture requiring intramedullary nail fixation of the tibia. Three months later, she sustained a mechanical fall causing further displacement of the right fibula, which was not repaired and resulted in chronic nonunion. She successfully completed a physical rehabilitation program and had been doing well until the time of presentation to the ED. The patient had been participating in a rehabilitation program for narcotic dependence that arose after her previous orthopedic surgery. She was otherwise healthy.

**Image 1.**
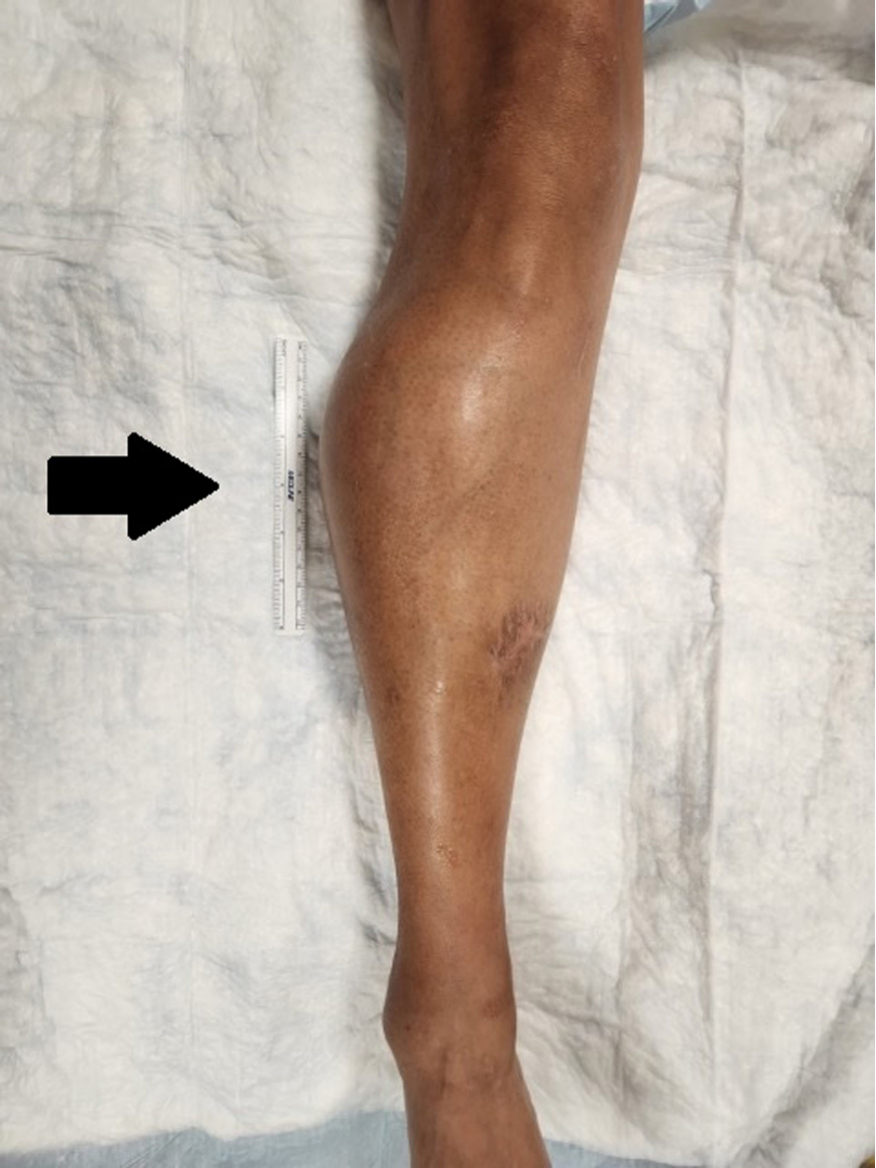
Anterior view of patient’s right lower leg with black arrow indicating area of tense and painful swelling.

Examination was remarkable for marked swelling of the anterolateral portion of the right lower extremity just distal to the knee (Image 1). The right lower leg compartments were firm, mildly compressible, tender, and nonpulsatile. Her right dorsalis pedis and posterior tibialis artery pulses were intact, and she had some mild numbness and paresthesias in the distribution of the superficial peroneal nerve distally. She did not have pain with passive ankle dorsiflexion or plantarflexion, and there were no overlying skin changes.

**Image 2.**
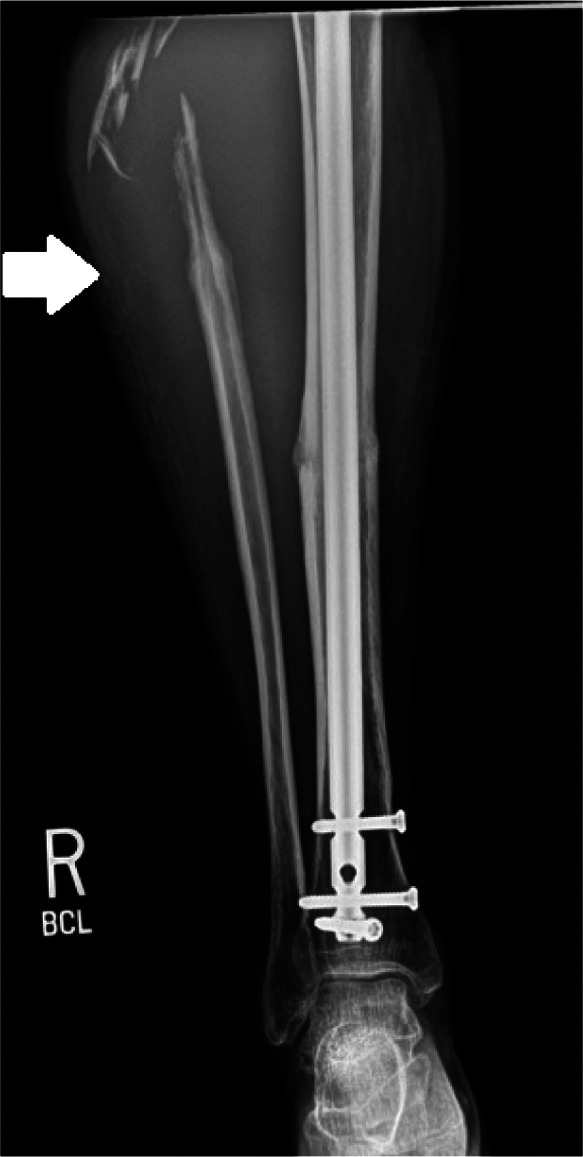
Initial anterior-posterior view of tibia/fibula on presentation, demonstrating unrepaired proximal fibular fragments with surrounding soft tissue swelling indicated by white arrow.

**Image 3.**
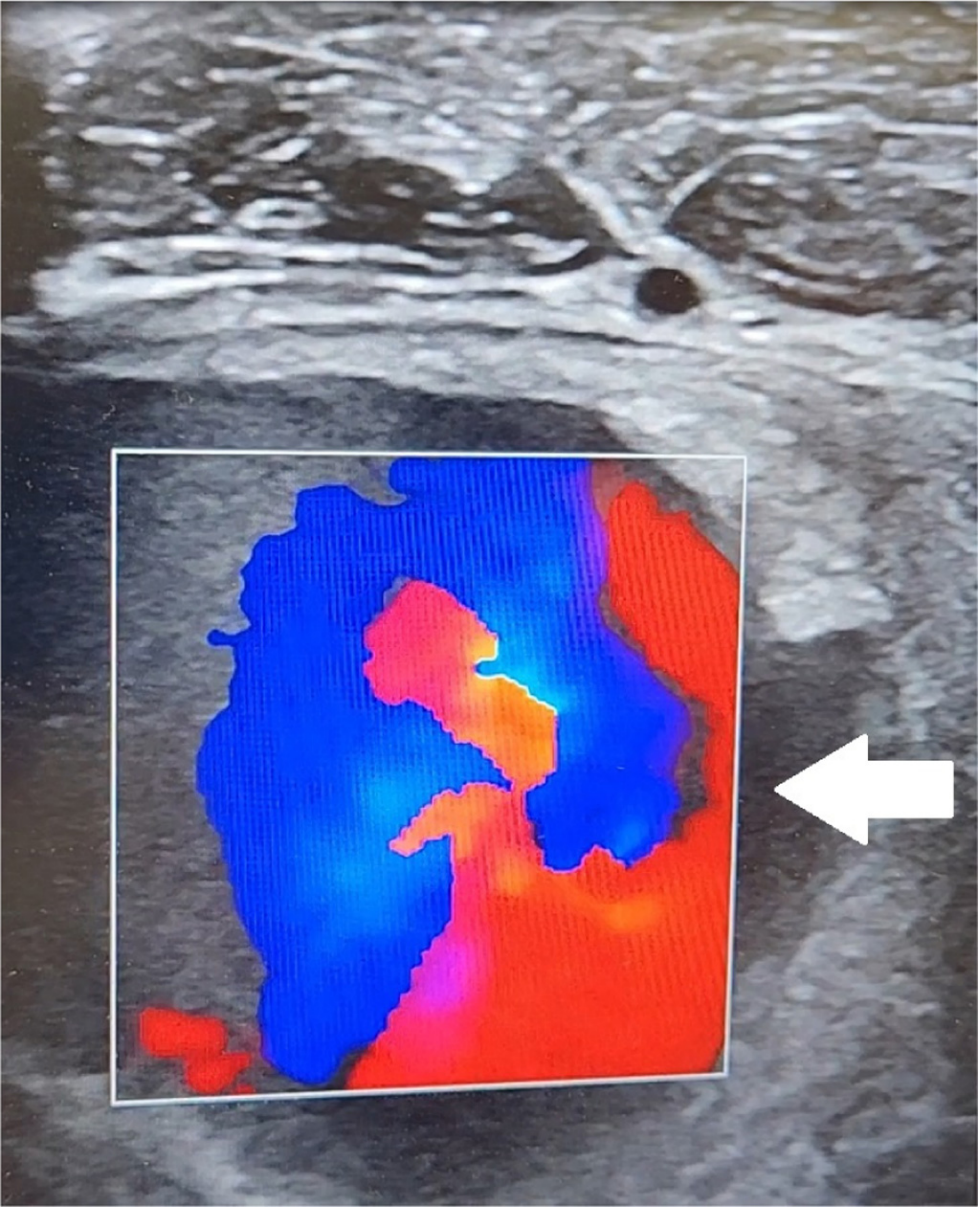
Point-of-care ultrasound demonstrating a 4 × 3 centimeter anechoic collection arising from the anterior tibial artery with to-and-fro color flow seen within the collection, consistent with a pseudoaneurysm as indicated by white arrow.

**Image 4.**
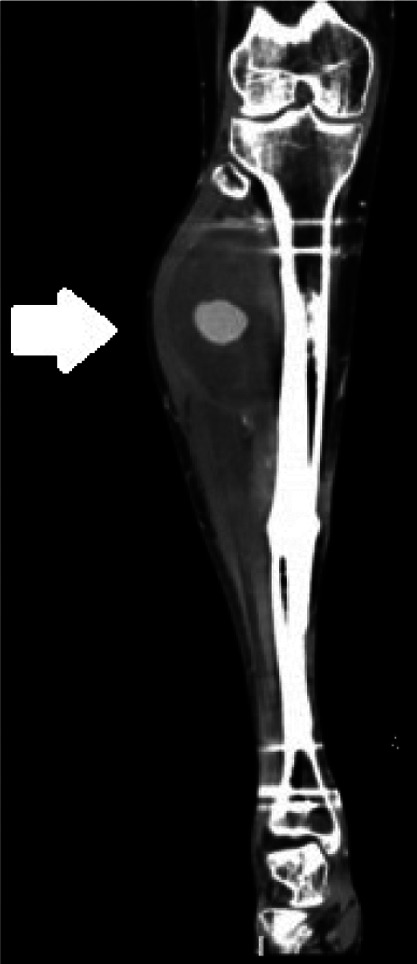
Coronal cut of computed tomography angiogram of the right lower extremity demonstrating a heterogeneous collection in the lateral, upper right calf, consistent with a hematoma. On the arterial phase, there is contained ballooning of contrast arising from the anterior tibial artery, consistent with a pseudoaneurysm in the area indicated by white arrow.

Radiographs demonstrated prior traumatic injury with hardware in place and marked soft tissue swelling in the lateral proximal calf (Image 2). Point-of-care ultrasound (POCUS) revealed pulsatile flow at the right anterolateral lower leg, suggestive of a pseudoaneurysm (Image 3). Formal ultrasound confirmed the presence of a three-centimeter pseudoaneurysm arising from the anterior tibial artery along the margin of an 11-centimeter hematoma in the upper, lateral right calf. This finding was also confirmed on computed tomography angiogram (Image 4).

Vascular surgery was consulted, and the patient underwent an open repair of the right anterior tibial artery pseudoaneurysm with evacuation of the hematoma and a four-compartment fasciotomy. Intraoperatively, the patient was noted to have jagged bone fragments from her fibular fracture in the pseudoaneurysm cavity, which were removed. Fasciotomy was also performed at that time because the patient had clinical signs of compartment syndrome, which included sensory deficit in addition to tense and swollen compartment. Her numbness resolved, and she was discharged home on postoperative day two.

## DISCUSSION

We describe the case of a patient presenting with a presumed post-traumatic pseudoaneurysm in the setting of chronic right fibular fracture with nonunion. The initial presentation of lower extremity pain and swelling with sensory deficit was concerning for compartment syndrome. The patient’s workout activity 24 hours prior to presentation was suspected to have caused right anterior tibial artery damage from the adjacent right fibular bone fragments. This ultimately resulted in pseudoaneurysm formation. Traumatic pseudoaneurysm has been previously described only rarely in case reports.^2^ ^,^ ^3^ Because compartment syndrome and pseudoaneurysm can present similarly, it is essential to discern vascular involvement. Fortunately, POCUS was performed and rapidly revealed pseudoaneurysm, which was confirmed upon formal ultrasound and computed tomography. Point-of-care ultrasound has become a mainstay of ED evaluations and in this case prevented catastrophe. Rapid identification of anechoic mass with definitive color demonstration of pulsatile component was achieved prior to decompression of the area. This facilitated appropriate vascular surgery consultation and controlled operative management. The insertion of a needle for measurement of compartment pressures in this patient with a swollen, tense extremity could have resulted in hemorrhage. We advocate for the use of POCUS to rapidly assess patients with similar presentations to rule out underlying vascular pathology such as psuedoaneurysms before proceeding with compartment pressure measurements.

## CONCLUSION

Traumatic pseudoaneurysms of the limbs are rare and should be included in the differential of limb swelling. Emergency physicians must be able to rapidly identify and initiate treatment for this potentially limb- threatening condition. This can easily be accomplished with point-of-care ultrasound, which has become an essential tool in emergency medicine. In this case, we were able to use POCUS to direct operative intervention preventing premature compartment pressure measurement, which could have led to massive hemorrhage.
